# Lung Function in African Infants: A Pilot Study

**DOI:** 10.1002/ppul.22965

**Published:** 2013-12-11

**Authors:** DM Gray, L Willemse, A Alberts, S Simpson, PD Sly, GL Hall, HJ Zar

**Affiliations:** 1 Department of Paediatrics and Child Health, Red Cross War Memorial Children's Hospital, University of Cape TownCape Town, South Africa; 2 Telethon Institute for Child Health Research, Centre for Child Health Research, University of Western AustraliaPerth, Australia; 3 The Queensland Children's Medical Research Institute, University of QueenslandBrisbane, Australia

**Keywords:** respiratory function test, South Africa, paediatric

## Abstract

**Background:**

The burden of childhood respiratory illness is large in low and middle income countries (LMICs). Infant lung function (ILF) testing may provide useful information about lung growth and susceptibility to respiratory disease. However, ILF has not been widely available in LMICs settings where the greatest burden of childhood respiratory disease occurs.

**Aim:**

To implement and evaluate a pilot study of ILF testing in a semi-rural setting in South Africa.

**Method:**

Infant lung function testing was established at a community hospital in South Africa. All measures were done in unsedated infants during sleep. Measurements, made with the infant quietly breathing through a face mask and bacterial filter, included tidal breathing (TBFVL), exhaled nitric oxide (eNO), and sulphur hexafluoride multiple breath washout (MBW) measures using an ultrasonic flow meter and chemoluminescent NO analyzer.

**Results:**

Twenty infants, mean age of 7.7 (SD 2.9) weeks were tested; 8 (40%) were Black African and 12 (60%) were mixed race. Five (25%) infants were preterm. There were 19 (95%) successful TBFVL and NO tests and 18 (90%) successful MBW tests. The mean tidal volume was 30.5 ml (SD 5.9), respiratory rate 50.2 breaths per minute (SD 8.7), and eNO 10.4 ppb (SD 7.3). The mean MBW measures were: functional residual capacity 71 ml (SD 13) and the lung clearance index 7.6 (SD 0.5). The intra-subject coefficient of variations (CV) of lung function measures were similar to published normative data for Caucasian European infants.

**Conclusion:**

In this study we demonstrate that unsedated infant lung function measures of tidal breathing, MBW, and eNO are feasible in a semi-rural African setting with rates comparable to those reported from high income countries. **Pediatr Pulmonol. 2015; 50:49–54.** © 2013 The Authors. Pediatric Pulmonology published by Wiley Periodicals, Inc.

## BACKGROUND

Lung development is incomplete at birth with much lung growth occurring during the first few years of life. Assessment of lung function longitudinally from birth may offer valuable information about the true burden, determinants, and outcomes of low lung function in early life. An objective measure of lung function is necessary to achieve this. Until recently infant lung function (ILF) testing has been largely restricted to isolated research institutions using custom made equipment. This made it difficult to compare results reducing generalizability among sites. Equipment was often bulky, testing moderately invasive, and almost exclusively done under sedation, which limited its use in well infants and particularly its use in community settings. Recent advances in technology, standardization of ILF measurement equipment and techniques through international expert collaboration has allowed the development of tests that are easily done in unsedated infants and have made ILF a more accessible and useful tool.^1^,^2^ ILF is now routinely used in high income countries as an epidemiological and clinical tool.^3^–^6^

The burden of acute and chronic respiratory disease is high in low and middle income countries (LMICs). Respiratory illness is a leading cause of early childhood death in LMICs.^7^,^8^ In addition chronic respiratory illness is increasingly common and associated with a substantial burden of disease in African children and indeed globally.^9^ Developing respiratory function measures that can be used in these settings to assess determinants of respiratory disease is desirable.

However little is known about ILF in African children, a region with one of the highest burdens of childhood respiratory disease.^9^,^10^ Infant lung function has not been previously undertaken in South Africa or any other African setting. We hypothesized that unsedated infant lung function measures, including tidal breathing, exhaled nitric oxide, and multiple breath washout testing, can be safely and successfully undertaken in a semi-rural setting in the Western Cape, South Africa. Hence, we aimed to demonstrate feasibility of ILF in this setting and to provide pilot data for a larger birth cohort study, the Drakenstein Child Lung Health Study (DCLHS).

## METHOD

### Ethics

The study was approved by the Faculty of Health Sciences, Human Research Ethics Committee, University of Cape Town (401/2009) and by the Western Cape Provincial Health Research committee. Written informed parental consent was obtained in the first language of the parent.

### Site Description

Infant lung function testing was established at Paarl hospital, a community hospital located in the semi-rural Drakenstein region. The hospital serves a low socioeconomic community of approximately 200,000 people; all births in the public sector (approximately 90% of all births in the area) occur at this hospital.

### Participants

Infants born between March and June 2012 at Paarl hospital, South Africa were eligible for enrolment. All infants in the Drakenstein area are born at this hospital. Infants born during enrolment period, who were healthy and whose parents gave consent were eligible for inclusion. Any infant with an acute or chronic illness was not included. Infant testing was booked for 6–10 weeks of age and was delayed until at least 2 weeks after any acute respiratory infection.

### Infrastructure Development and Training

An international collaboration was established between pediatric pulmonologists in South Africa and ILF specialists in Australia. The local investigator and respiratory technologist were trained in Perth, Australia during Nov to Dec 2011 in ILF testing techniques. Training included the set up, calibration, data collection and analysis of the tidal breathing measures (TBFVL), exhaled nitric oxide (eNO), and multiple breath washout (MBW) techniques. A second technologist was trained on-site during the set up period. Standard operating procedures were established with oversight from the international collaborators, who also provided ongoing supervision and training through regular teleconference. During this period early studies were over-read by the experts in Perth with feedback to the study team. The equipment used was the EcoMedics ExhalyzerD with ultrasonic flow meter and CLD88 NO chemoluminescent analyzer (EcoMedics, Duernten, Switzerland). An initial introduction to the equipment was provided during the set up by the company; the local team was then assisted through email and teleconference communication during the first months for trouble shooting. Supply chains for consumables including SF6 testing and NO calibration gas mixtures were successfully established.

### Infant Lung Function

Infant lung function was tested between 1 and 3 months of age. All tests were done by the same testing team, which consisted of a trained respiratory technologist and a professional nurse, supervised by a pediatric pulmonologist. Testing was measured in unsedated infants during behaviorally defined quiet natural sleep.^11^ If the child woke during a measurement the child was settled and testing restarted when the child was in quiet sleep. Measurements were taken with infants lying supine with their head in the midline using a size 0 round infant silicone placed over the nose and mouth. The effective deadspace of the mask (5 ml) was determined as the water displacement volume of the mask on a curved surfaced and applying similar pressure to the mask as used during an actual test. The water displacement volume was then halved to determine the effective mask deadspace as recommended previously^1^,^12^ and as published by our group.^13^,^14^ Testing was performed on commercially available equipment that conformed to the ATS/ERS ILF equipment standards.^1^ Flow was measured using a commercially available ultrasonic flow meter (Ecomedics AG, Duernten Switzerland). A dead space reducer (size 1) was used to reduce the internal dead space of the flow meter to 1.2 ml. A disposable bacterial filter (Spirette™, EcoMedics AG, Duernten, Switzerland) surrounded the dead space reducer. A bias flow of 200 ml sec^−1^ was used. During data collection, flow-volume loops were observed to ensure there was no leak. Data were recorded at ambient temperature, pressure, and saturation with flow and volume being converted to body temperature and pressure, saturated conditions during data analysis. Leak was defined as volume drift of >3 ml sec^−1^ and data excluded in these cases. Measurements were collected in the following order: TBFVL and simultaneous eNO for 90 sec, followed by three acceptable MBW measurements where possible. Data analysis, detailed for each test below, was independently repeated by experts in Perth to assure quality control. Test analysis was blinded with neither of the two sites, Perth and Drakenstein, having reference to the other score.

*Tidal breathing recordings (TBFVL)* were obtained during quiet tidal breathing at least 30 sec after initial mask placement. Recordings were analyzed if >30 consecutive regular breaths of tidal breathing were recorded during a 90-sec epoch and according to international guidelines.^15^ Mean tidal breathing measures were calculated using analysis software (Wbreath v3.2.0, Ndd Medizintechnik AG, Zurich, Switzerland) as reported previously.^16^,^17^ Outcome parameters included respiratory rate, tidal volume, minute ventilation, mean tidal inspiratory flow, mean tidal expiratory flow, and the ratio of time to peak tidal expiratory flow/expiratory time as a description of the shape of the tidal breathing flow volume loop.

*Exhaled nitric oxide (eNO)* measurements were recorded simultaneously with the TBFVL and according to international guidelines and as previously published by our group.^17^,^18^ Exhaled NO was an online measurement with a rapid-response, chemoluminescence analyser (CLD 88 Exhalyzer, Ecomedics AG, Duernten, Switzerland). Testing used NO free air for inspiration avoiding contamination from ambient NO. Exhaled NO was calculated breath by breath during the third quartile of expiration and the mean eNO calculated from a minimum of 30-recorded breaths using the analysis software (Wbreath v3.2.0, Ndd Medizintechnik AG, Zurich, Switzerland). The outcome parameters recorded included the eNO and the NO output (eNO × corresponding expiratory flow).

*Multiple breath washout (MBW)* testing was performed using 5% SF6 as a tracer gas and ultrasonic flow meter (Spiroson®, Ecomedics AG, Duernten, Switzerland) with acquisition and analysis software (Wbreath v3.2.0, Ndd Medizintechnik AG, Zurich, Switzerland) as reported by our group^13^ and in accordance with recent recommendation for the measurement of MBW.^19^ The washout period began after a ten breath equilibrium period was obtained at the end of the tracer gas wash in. The washout continued until the tracer gas was eliminated from the lungs. The outcome parameters included the functional residual capacity (FRC) and the lung clearance index (LCI; cumulative expired volume/FRC) and moment ratios (M_1_/M_0_ and M_2_/M_0_). The recordings were defined as acceptable for analysis if they occurred during quiet sleep with no sighs within 10 breaths of the wash-in plateau or 10 breaths after the SF6 concentration has returned to baseline. The process was repeated to obtain three successful recordings. If the child woke testing was restarted when infant went back to sleep, provided the parent was happy to wait. Analysis of the recordings using the analysis software (Wbreath v3.2.0, Ndd Medizintechnik AG, Zurich, Switzerland), included the use of optimized temperature and deadspace model.^16^ Flow and volume were converted to body temperature and pressure, saturated conditions during data analysis. The mean FRC and LCI value of the three tests was reported or the mean of two tests if the mean was within 10% of the lower value.

## STATISTICAL ANALYSIS

Descriptive statistics were performed using STATA 11 for windows (STATA Corporation, College Station). Data are presented as mean and standard deviation (SD), median, and interquartile range (IQR) and the 95% limits of agreement, which was defined as the mean difference ±1.96 SD. The intra-subject coefficient of variation (CV) was calculated as the ratio of each parameter standard deviation over the parameters mean per study participant. For FRC and LCI this was calculated from the mean and standard deviation of the MBW tests collected. The agreement between observers' results from the two sites was presented as the mean difference and SD, and minimum and maximum values.

## RESULTS

The demographics of included infants are summarized in Table 1.Twenty infants with a mean age of 7.7 (range 3.4–14) weeks consented for participation. Five (25%) were preterm, defined as born before 37 weeks post gestational age. Of the infants tested one TBFVL and one eNO test was excluded due to poor quality trace on analysis (less than 30 regular breaths selected), thus there were 19 (95%) successful TBFVL and eNO tests. Two MBW tests were excluded as one child woke up and could not complete the test and in the second the mean of the two tests obtained was not within 10% of the lower value; there were therefore 18 (90%) successful MBW tests. The median time taken per infant test was 22 min (range 6–64). Six (30%) infants woke during testing and had to be settled for a second measurement. One infant did not sleep and required rebooking on another day to complete testing. The median length of lung function visit, including waiting time for infants to sleep was 159 min (range: 27–320).

**TABLE 1:** Demographics of Infants (n = 20)

Characteristic at study date	Mean (SD)	Med (min; max)
Age (weeks)^1^	7.7 (2.9)	6.6 (3.4; 14)
Weight (kg)	4.6 (0.99)	4.5 (3; 6.2)
Height (cm)	51.5 (3.1)	50.5 (47; 57)
Time taken for testing (min)	23 (12)	22 (6; 64)
Gestational age at birth (weeks)	37 (1.8)	37 (33; 39)

	*N (%)*	
Maternal smoking in pregnancy	5 (25)	
Female gender	13 (65)	
Ethnicity		
Black African	8 (40)	
Mixed race	12 (60)	

1 Age corrected to 37-week gestation for preterm infants.

Tidal breathing and MBW results are shown in Table 2. The mean (SD) difference between the blinded analyses of the Perth and Drakenstein sites for FRC in ml was 0.17 (0.5), range: 0–2.2 and LCI 0.05 (0.1); range: 0–0.5. The intra-subject coefficient of variation (CV) demonstrates the variability for each outcome and ranges from 6.5% for minute ventilation (V′E) to 19.7% for the ratio of time to peak tidal expiratory flow over the total expiratory time (PTEF/TE).

**TABLE 2:** Tidal Breathing, Multiple Breath Washout and Exhaled Nitric Oxide Measurements

Tidal breathing	Number (n)	Mean (SD)	Median (IQR)	95% limits of agreement	Median (IQR) intra-subject CV^1^
Tidal volume (ml)	19	30.5 (5.8)	30.2 (25.3; 34.4)	18.9 to 42.1	6.8 (6; 7.9)
Respiratory rate breaths per min	19	49.4 (8.4)	49.6 (42.9; 54.8)	41 to 65.9	7.7 (6.5; 9.3)
Minute ventilation (ml/min)	19	1,487.4 (319.8)	1,433.5 (1,275.3; 1,634.7)	860.6 to 2,114.2	6.5 (4.6; 8.3)
PTEF/TE^2^	19	37 (11.5)	35 (27; 44.8)	7.46 to 34.26	19.7 (15.6; 25.2)
MTIF^3^	19	54.8 (10.2)	54.3 (50; 57.3)	34.9 to 74.7	6.6 (5.4;10)
MTEF^4^	19	46 (12)	45.8 (36.2; 52)	22.3 to 69.7	7.8 (6.2; 10)

Multiple breath washout	Number (n)	Mean (SD)	Median (IQR)	95% limits of agreement	Median (IQR) intra-subject CV^1^
FRC^5^ (ml)	18	70.7 (13)	66.3 (61; 83)	45.2 to 96.2	6.8 (4.5; 8.8)
LCI^6^	18	7.6 (0.5)	7.5 (7.3; 7.8)	6.6 to 8.6	3.6 (1.6; 6.4)
Moment ratio m1/m0	18	1.5 (0.1)	1.5 (1.3; 1.6)	1.3 to 1.7	
Moment ratio m2/m0	18	5.6 (0.8)	5.8 (4.2; 6.6)	4 to 7.2	

Exhaled nitric oxide	Number (n)	Mean (SD)	Median (IQR)	95% limits of agreement	Median (IQR) intra-subject CV^1^
NO ppb	19	10.4 (7.3)	9.7 (3.4; 17.2)	−4 to 24.7	4.2 (3.3; 5.5)
NO output (mcl/sec)	19	7.8 (2.2)	7.2 (6.2; 9.1)	3.5 to 12.1	2.3 (0.8;3)

1 Coefficient of variation.

2 Ratio of time to peak tidal expiratory flow over total expiratory time.

3 Mean tidal inspiratory flow.

4 Mean tidal expiratory flow.

5 Functional residual capacity.

6 Lung clearance index.

The multiple breath washout test results, Table 2, include measures of lung volume, the functional residual capacity (FRC) and measures of ventilation inhomogeneity the lung clearance index (LCI) and moment ratios. The intra-subject CV was 6.8% for the FRC and 3.6% for the LCI. The exhaled nitric oxide results, Table 2, include exhaled NO and NO output.

## DISCUSSION

This study describes the successful implementation of an ILF testing site in a semi-rural setting in South Africa. This is the first time ILF testing, predominantly used in specialized centers in high-income settings, has been used in Africa.

The success rates for testing were similar to those reported by Fuch's et al.^20^, who recorded an 85% testing success for TBFVL measures, 76% for eNO, and 59% for MBW testing in 342 healthy unsedated European infants at 4–9 weeks of age. Although the equipment and testing procedures used in this study were similar, the Fuchs study used a longer tidal breathing epoch of 100, compared to 30, consecutive regular breaths during quiet sleep. This would have prolonged testing and possibly reduced the chance of successfully recording all measures before infants woke.

Successful implementation depended on several key factors. These included close and ongoing collaboration with international experts experienced in ILF testing, initial training in an experienced laboratory, back up technical support from the supplier of the equipment, sufficient resources for equipment, training and staffing, a defined research space and dedicated, well trained staff onsite. The initial training of a local technologist in the overseas ILF center allowed for the training of further local technologists. This collaboration ensured strict supervision of data quality and allowed double analysis of data. This collaborative work is possible with standard internet connectivity and did not necessarily require teleconference facility.

The median time of testing during this study was 22 min (IQR 6; 64). This only included active testing time. Six (30%) of the infants required resettling and a second attempt at testing hence the total visit time (median [range]: 159 min [27–320]) was long in these infants. Thus successful completion of unsedated infant lung function testing can be time consuming. Importantly, no sedation was used, thus the measurements reflect physiological lung volumes during sleep. Furthermore, this makes the procedure especially safe and reduced the need for post test observation and monitoring.

The results of the testing were comparable to the normative values for European infants published by Fuchs et al.^20^ (Table 3). This comparison is however limited by the pilot studies small sample size, and should be confirmed in a larger cohort from a similar South African community. In addition the pilot cohort included infants born prematurely, a known risk factor for early low lung function.^21^ However, none of our infants were born less than 33 weeks gestation and mean gestation was 37 weeks. Twenty-five percent of infants' mothers smoked during pregnancy. Maternal smoking is a known risk factor for low lung function at birth.^22^,^23^ The results of this study cannot be used to inform normative ranges for African children given the small sample size of this pilot and the inclusion of infants at risk of low lung function.

**TABLE 3:** Drakenstein Infant Lung Function Compared European Birth Cohort

	Drakenstein pilot study (N = 20)		European study (N = 296)^18^	
		
	Mean (SD)	Median (IQR) intra-subject CV^1^	Mean (SD)	Median (IQR) intra-subject CV^1^
**Tidal breathing N successful test (%)**	**19 (95)**		**285 (96)**	
Tidal volume (ml) mean (SD)	30.5 (5.8)	6.8 (6; 7.9)	32.4 (5.5)	8.6 (7.1; 10.8)
Respiratory rate breaths per min, mean (SD)	49.4 (8.4)	7.7 (6.5; 9.3)	45.2 (10.5)	9.1 (7.4; 11.3)
Minute ventilation (ml/min), mean (SD)	1,487.4 (319.8)	6.5 (4.6; 8.3)	1,420 (277)	7.5 (6.1; 10.1)
PTEF/TE^2^	37 (11.5)	19.7 (15.6; 25.2)	34.8 (10.7)	23.8 (20.2; 28.4)
**Multiple breath washout N successful tests (%)**	**18 (90)**		**201 (68)**	
FRC^3^ (ml), mean (SD)	70.7 (13)	6.8 (4.5; 8.8)	102 (16)	6.3 (4.4; 8.3)
LCI^4^	7.6 (0.5)	3.6 (1.6; 6.4)	6.75 (0.6)	5.8 (3.6; 8.0)
**Exhaled nitric oxide N successful tests (%)**	**19 (95)**		**261 (88)**	
NO^5^ ppb	10.4 (7.3)	4.2 (3.3; 5.5)	14.3 (6)	10.3 (6.4; 15.7)
NO output (mcl/sec)	7.8 (2.2)	2.3 (0.8; 3)	6.3 (2.5)	12.4 (8.9–18.1)

1 Coefficient of variation.

2 Ratio of time to peak tidal expiratory flow over total expiratory time.

3 Functional residual capacity.

4 Lung clearance index.

5 Nitric oxide.

The results had acceptable variability, as expressed by the intra-subject CV, supporting the feasibility of the proposed larger project. In the tidal breathing measures the CV for each parameter was less than 10% except for the tPTEF/TE, which was 19.7%. This is consistent with previously published results of similar testing in unsedated infants.^20^,^24^ The mean FRC in this study 70.7 (SD 13) was lower than the European study, 102 (SD 16). The LCI was higher, 7.6 (SD 0.5) compared to 6.7 (SD 0.6) in the European study. The infants in the South African cohort were an average of 2 weeks older than the European cohort. The difference in FRC and LCI may relate to the different ethnicity or socioeconomic status of this group, factors that have been previously reported to effect lung function measurements in young children.^25^,^26^ A larger cohort is needed to confirm this and investigate possible associations. The mean eNO in this study of 10.4 (SD 7.3) was lower than the 14.3 (SD 6) than that reported by Fuchs et al. However, the sample size is too small to make meaningful comparisons and further study with a larger sample is required.

In conclusion, this pilot study has described the development of technical and clinical capacity in ILF testing in a LMIC setting. This study provides reliable data for supporting the use of these measures in a prospective birth cohort study, the Drakenstein Child Lung Health Study. This technique has the potential to be a useful tool in obtaining valuable information on early lung growth, function and the development of acute and chronic lung diseases in children living in high burden settings including Africa.
